# Monthly mobility inferred from isoscapes and laser ablation strontium isotope ratios in caprine tooth enamel

**DOI:** 10.1038/s41598-021-81923-z

**Published:** 2021-01-26

**Authors:** N. Lazzerini, V. Balter, A. Coulon, T. Tacail, C. Marchina, M. Lemoine, N. Bayarkhuu, Ts. Turbat, S. Lepetz, A. Zazzo

**Affiliations:** 1 Archéozoologie, Archéobotanique: Sociétés, Pratiques et Environnements (AASPE), Muséum National d’Histoire Naturelle, Sorbonne Université, Centre National de La Recherche Scientifique (CNRS), CP 56, 55 rue Buffon, 75005 Paris, France; 2 Laboratoire de Géologie de Lyon, Terre, Planètes, Environnement (LGLTPE), École Normale Supérieure Lyon, Université Lyon 1, Centre National de la Recherche Scientifique (CNRS), 46 Allée d’Italie, 69342 Lyon Cedex 07, France; 3 Centre d’Écologie et des Sciences de la Conservation (CESCO), Muséum National d’Histoire Naturelle, Centre National de la Recherche Scientifique (CNRS), Sorbonne Université, CP 135, 57 rue Cuvier, 75005 Paris, France; 4 Centre d’Ecologie Fonctionnelle et Evolutive (CEFE), Centre National de la Recherche Scientifique (CNRS), École Pratique des Hautes Études (EPHE), Institut de Recherche Pour le Développement (IRD), Université Paul Valéry Montpellier 3, 34090 Montpellier, France; 5 Bristol Isotope Group, School of Earth Sciences, University of Bristol, Wills Memorial Building, Queen’s Road, Bristol, BS8 1RJ UK; 6 Institut Français de Recherche Sur l’Asie de l’Est (IFRAE), Institut National des Langues et Civilisations Orientales (Inalco), Université de Paris, Centre National de la Recherche Scientifique (CNRS), 2 rue de Lille, 75007 Paris, France; 7 Institute of Archaeology, Mongolian Academy of Sciences, Ulaanbaatar, Mongolia

**Keywords:** Animal migration, Stable isotope analysis, Biogeochemistry, Environmental sciences

## Abstract

Strontium isotopic analysis of sequentially formed tissues, such as tooth enamel, is commonly used to study provenance and mobility of humans and animals. However, the potential of ^87^Sr/^86^Sr in tooth enamel to track high-frequency movements has not yet been established, in part due to the lack of data on modern animals of known movement and predictive model of isotope variation across the landscape. To tackle this issue, we measured the ^87^Sr/^86^Sr in plant samples taken from a 2000 km^2^ area in the Altai Mountains (Mongolia), and the ^87^Sr/^86^Sr in tooth enamel of domestic caprines whose mobility was monitored using GPS tracking. We show that high-resolution, sequential profiles of strontium isotope composition of tooth enamel reliably reflect the high-frequency mobility of domestic livestock and that short-term residency of about 45 days can be resolved. This offers new perspectives in various disciplines, including forensics, ecology, palaeoanthropology, and bioarchaeology.

## Introduction

The analysis of radiogenic strontium isotope ratios (^87^Sr/^86^Sr) in tooth enamel is commonly used to study provenance and mobility of modern-day humans in forensics^1,2^, past humans in palaeoanthropology^3,4^, and domestic and wild mammals in ecology and archeology^5–8^. In all cases, the rationale is the following. Variations in the radiogenic ^87^Sr/^86^Sr in the landscape are associated with the age, chemical composition, and weathering rates of the underlying geological substrate because ^87^Sr is the stable product of the radioactive ^87^Rb. Any contribution of mass-dependent fractionation to variability of ^87^Sr/^86^Sr is removed by their normalization to a constant stable ^88^Sr/^86^Sr; therefore the primitive bedrock ^87^Sr/^86^Sr are conserved up the trophic chain, without any possible change. Mammal tooth enamel is no exception, and because it grows incrementally and is not remodeled once fully mineralized, the ^87^Sr/^86^Sr in tooth enamel reflects that of the bedrock at the moment of mineralization. The tooth enamel hence holds a ^87^Sr/^86^Sr time record that can be documented through sequential sampling along the direction of tooth growth^9^.

Although it is, in theory, possible to reconstruct rapid mobility using high-resolution ^87^Sr/^86^Sr time series in tooth enamel, this has never been formally demonstrated. Several reasons can be put forward to explain this. First, there is a lack of studies using individuals whose mobility is known with precision. Second, the kinetics of Sr incorporation (deposition and maturation) in tooth enamel during mineralization are still not well understood^10^. Targeting the innermost enamel layer (< 20 μm thickness) has been proposed as the best strategy for measuring any geochemical proxy^11–15^. This zone is a good prospect because it mineralizes faster than middle and outer enamel^11^, and thus minimizes the attenuation of environmental variations recorded in this tissue. However, this layer remains difficult to isolate precisely for analysis. In theory, this could be achieved using laser ablation as this approach allows high-resolution analysis of the ^87^Sr/^86^Sr ratio and the reconstruction of ^87^Sr/^86^Sr time series^3,16–18^.

Finally, the reconstruction of geographical origin and mobility also relies on an acute knowledge of the local bioavailable ^87^Sr/^86^Sr. To this end, isoscapes, which are predictive models of isotope variations across a landscape, have been generated using either geostatistical approaches^19^, multi-source mixing models^20^, machine learning^21^, or discrete entities^22^. Isoscapes can be constructed by taking into account the bedrock lithology only, but this has limitations, notably due to preferential weathering of minerals with distinct ^87^Sr/^86^Sr; influence of atmospheric deposition (i.e. dust, precipitation, sea spray); and anthropogenic influences, such as fertilizer or air pollution^23–28^. Constructing isoscapes by measuring the plant ^87^Sr/^86^Sr has been demonstrated as the most reliable method for determining local bioavailable ^87^Sr/^86^Sr^22,26,29^.

Here, we test the ability of ^87^Sr/^86^Sr to detect high-frequency mobility of domestic animals living in the Altai mountains of western Mongolia. To do this, we compared high-resolution ^87^Sr/^86^Sr time series in domestic caprine tooth enamel with predictions derived from a plant ^87^Sr/^86^Sr isoscape of the study area. We monitored the animals’ movements for more than 2 years using GPS collars, allowing us to precisely document their mobility. We used this information to predict intra-tooth ^87^Sr/^86^Sr from the isoscape. To this end, we (i) measured ^87^Sr/^86^Sr in plants from 156 sampling sites; (ii) constructed an isoscape of the bioavailable ^87^Sr/^86^Sr of the study area; (iii) constructed the theoretical bioavailable ^87^Sr/^86^Sr time series using the GPS data and the ^87^Sr/^86^Sr isoscape for four herds; (iv) converted the bioavailable ^87^Sr/^86^Sr time series into enamel ^87^Sr/^86^Sr time series using the tooth enamel growth model of Passey and Cerling (2002), leading to four predicted time series of ^87^Sr/^86^Sr variations in enamel; (v) measured the ^87^Sr/^86^Sr in the inner enamel layer of teeth using laser-ablation multi-collector inductively coupled plasma mass spectrometry (LA-MC-ICPMS); (vi) optimized the cross-correlation between the associated 4 predicted and 11 measured time series. The synchronicity of ^87^Sr/^86^Sr variations in the predicted and measured time series was used to discuss the ability of ^87^Sr/^86^Sr to detect high-frequency mobility of the animals.

## Results

### Plant ^87^Sr/^86^Sr and bioavailable ^87^Sr/^86^Sr isoscape

**Figure 1:**
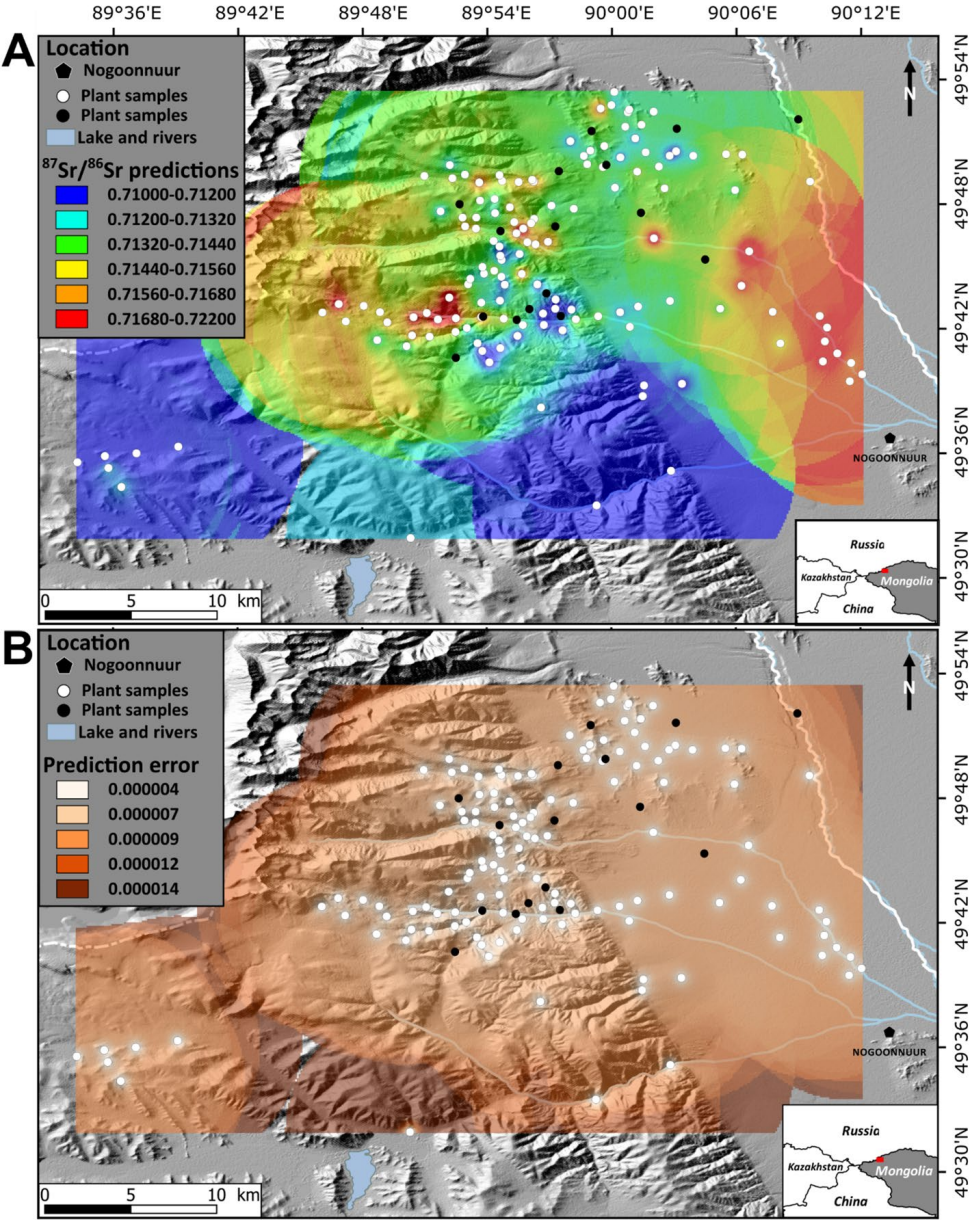
Bioavailable ^87^Sr/^86^Sr of the study area, near the village of Nogoonnur, western Mongolia. ^87^Sr/^86^Sr isoscape (**A**) and its associated predicted error (**B**) were created using QGIS 3.2 Bonn^51^ with the predictive model of bioavailable ^87^Sr/^86^Sr built with the kriging function in ArcGIS 10.0 (Environmental Systems Research Institute)^52^. White circles represent the bioavailable ^87^Sr/^86^Sr sampling localities used to create the model. Black dots represent the 16 randomly selected sampling locations that were extracted prior to model development and later used to test the model predictions. Colors indicate areas of lowest bioavailable ^87^Sr/^86^Sr (blue) to highest bioavailable ^87^Sr/^86^Sr (red) for the top panel and lowest predictive error (light beige) to highest predictive error (brown) for the bottom panel.

The ^87^Sr/^86^Sr in the plant samples ranged between 0.71007 and 0.72340, with an average of 0.71456 ± 0.00537 (± 2SD, *N* = 156) (SI Appendix, Table S1; Fig. S1A). The study area exhibited a wide range (0.01333) in bioavailable ^87^Sr/^86^Sr, 5 times higher than at Köşk Höyük (Central Turkey, 0.0025^30^) and 26 times higher than at Oldupai Gorge (Tanzania, 0.00052^31^), two areas of similar size (roughly 2000 km^2^). There was no significant relationship between the plant ^87^Sr/^86^Sr and geographic variables (i.e. latitude, longitude, and altitude; SI Appendix, Fig. S2A–C). An ordinary kriging method was used to create the Sr isoscape of the study area. The main advantage of this method over other interpolation methods, is that it takes into account both the distance and the degree of variation between known data points when estimating values in unknown areas and that predictions are based on a spatial statistical analysis of the data. Detailed results are given in SI Appendix (Supplementary Information Text; Table S2; Figs. S3 and S4). The ^87^Sr/^86^Sr values predicted by the isoscape ranged from 0.71012 to 0.72251, with a mean value of 0.71457 ± 0.00530 (± 2SD). The isoscape highlighted the spatial heterogeneity of the study area, with the lowest values in the southern part and the highest values in the eastern and western parts (Fig. 1A). The standard error from the model predictions was evenly distributed and started increasing 6–7 km away from the sample locations (Fig. 1B).

### Bioavailable ^87^Sr/^86^Sr time series inferred by GPS monitoring

We monitored the movements of eight animals (four sheep and four goats) by means of GPS devices over a period of 29 months (SI Appendix, Table S3). Their range overlapped the plant sampling sites (SI Appendix, Fig. S1B). The Lavielle’s segmentation of individual trajectories indicated that the mobility patterns of caprines within the same herd were similar and that they differed from those of the other herds (SI Appendix, Tables S4 and S5; Fig. S5). Three herds (belonging to herders Jk, Kb, and Kj) displayed more or less an East–West mobility and exploited both the eastern depression (1451–1684 m asl) and the western mountain pastures (2540–3102 m asl). In contrast, herder Dl’s animals remained at an elevated altitude of 2364 ± 130 m asl throughout the study period (SI Appendix, Fig. S1B). This was associated with a smaller grazing area (12.5 km^2^ for Dl versus 18.9–22.3 km^2^ for the other three herders) and a lower yearly mobility (30 km for Dl versus 161 ± 98 km for the other three herders). The herders nomadized between 8 (Dl) and 13 (Jk) times per year during the study period and used between 5 (Dl) and 11 (Kj) different campsites; the average distance traveled during each nomadization ranged from 4 (Dl) to 17 (Jk) km (Table S4).

To predict daily bioavailable ^87^Sr/^86^Sr for a given herd based on its GPS location, we extracted the ^87^Sr/^86^Sr from the isoscape raster. For each herd, we then predicted the daily bioavailable ^87^Sr/^86^Sr value by calculating the average of the different bioavailable ^87^Sr/^86^Sr values for each day. Each herd exhibited temporal variations in bioavailable ^87^Sr/^86^Sr, with peaks occurring mostly during the spring and fall seasons (SI Appendix, Fig. S6). Most temporal variations were common to all herds, but some of them were characteristic of only some of them (e.g. the 2017 winter variations for Dl’s and JK’s herds). Sr isotope ratios remained generally stable during 2 months, which is the duration of the short spring and fall seasons, but some other levelling off were shorter, i.e. the 2017 spring levelling off for Dl’s herd, which lasted only 1 month. Most of these temporal variations in Sr isotope ratios corresponded to a nomadization and to a change of pasture by the herder and their herd between two isotopically different areas and extended as long as residence at a given camp lasted. For Dl, the fast decrease during winter 2017 can be linked to the nomadization from camp B to camp A (SI Appendix, Table S5; Fig. S5).

### Predicted ^87^Sr/^86^Sr time series in tooth enamel

We then converted the bioavailable ^87^Sr/^86^Sr time series into predicted ^87^Sr/^86^Sr in tooth enamel using Passey and Cerling’s model^32^. Originally developed for carbon isotopes, this model describes how the isotopic signal is time-averaged in enamel during the mineralization process. Overall, the effect of the mineralization process on the output variations of ^87^Sr/^86^Sr in tooth enamel resulted in the dampening of about 56% of the amplitude of variations of the input bioavailable ^87^Sr/^86^Sr (SI Appendix, Table S6; Fig. S6).

### Measured ^87^Sr/^86^Sr time series in tooth enamel

The Sr voltage (approximated by the ^88^Sr voltage) ranged from ~ 0.4 to ~ 5 V (SI Appendix, Fig. S7). There was no increase in ^87^Sr/^86^Sr associated to ^88^Sr decrease, which has been suggested to be symptomatic of enhanced influence of polyatomic Ca-P-O/Ar-P-O interferences at low Sr intensities^18,33–35^. The SRM-1400 bracketing standard had a Sr concentration of 250 ppm and exhibited a ^88^Sr voltage of 0.5 V and a reproducibility of 214 ppm (2SD, *N* = 50). This is more than 60 times lower than the range in plant ^87^Sr/^86^Sr of the study area. While the precision of this technique is lower than the resolution of MC-ICP-MS and thermal ionization mass spectrometry (TIMS) of purified strontium solution analyses, it is however, large enough to interpret variations of the bioavailable ^87^Sr/^86^Sr in terms of animal mobility.

**Figure 2:**
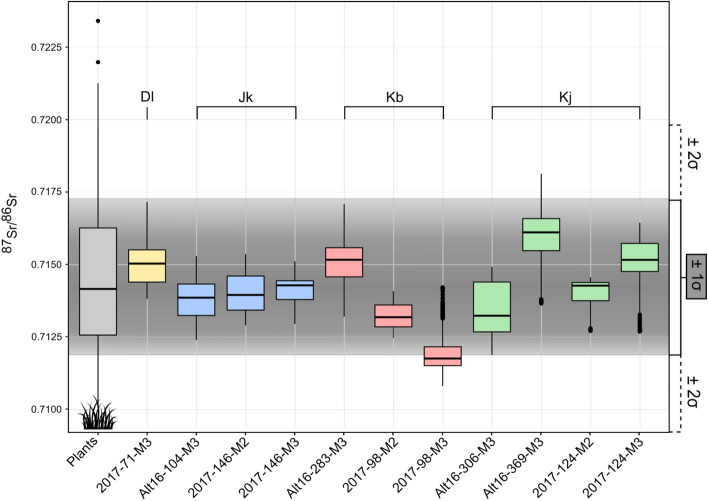
Sr isotope ratios of modern caprine teeth from western Mongolia. The horizontal brackets delineate the animals belonging to the same herder. Bio-available Sr isotope ratios (estimated in plants—gray boxplot) are reported for comparison. The gray shading represents the mean ± 1 SD of plant ^87^Sr/^86^Sr.

The ^87^Sr/^86^Sr in caprine teeth (*N* = 11) was measured along a continuous raster as close as possible to the innermost enamel layer. On average, the enamel ^87^Sr/^86^Sr was 0.71414 ± 0.00210 (± 2SD), falling within the range of bioavailable strontium predicted by the isoscape (Fig. 2 and SI Appendix, Table S6). The average ^87^Sr/^86^Sr, however, differed among individuals and ranged from 0.71200 ± 0.00087 (± 2SD) for Kb-98-M3 to 0.71602 ± 0.00198 (± 2SD) for Kj-369-M3. Intra-individual variability was 0.00143, ranging from 0.00087 (Kb-98-M3) to 0.00198 (Kj-369-M3). Large and significant inter-individual differences (ANOVA, p < 2.2e^−16^) within the same herd were observed, ranging from 0.00226 (Kj) to 0.00311 (Kb), but a smaller inter-individual difference of 0.00033 (Kb) was found within the same herd. Significant differences were observed in the ^87^Sr/^86^Sr means and medians (ANOVA, p < 2.2e^−16^) and in variance (Bartlett, p < 2.2e^−16^) between herds. Kb’s herd (*n* = 3) had the lowest mean value (0.71344 ± 0.00311, ± 2SD), while Kj’s herd (*n* = 4) had the highest mean value (0.71465 ± 0.00226, ± 2SD).

### Comparison of the predicted and measured time series

To compare measured and predicted values, it was first necessary to anchor the measured ^87^Sr/^86^Sr profiles in enamel to time. This required determining the date of birth (DOB) of the animals, the onset of mineralization of the second and third molars (M2 and M3), and the tooth growth rates of these molars. Based on interviews with the herders, a DOB of March 31 was set for all individuals, with an uncertainty of ± 1 month. An average onset of mineralization of 0.3 month and 10.3 months after birth was estimated for M2 and M3, respectively, associated with an uncertainty of approximately 1 month and 2–4 months for M2 and M3, respectively^36^. Finally, a constant and an exponentially decreasing growth rate were calculated for M2 and M3^37^. For three animals, both their M2 and M3 were analyzed (2017-98, 2017-146, and 2017-124). In these cases, the end of the M2 ^87^Sr/^86^Sr profiles joined up well with the beginning of the M3 ^87^Sr/^86^Sr profiles, leading to a continuous ^87^Sr/^86^Sr time series of more than 2 years.

Taken together, our analyses led to 4 predicted tooth enamel ^87^Sr/^86^Sr time series and 11 measured tooth enamel ^87^Sr/^86^Sr time series, which we compared accordingly. Time uncertainties were associated with the results, especially about the DOB of the animals and the growth rate of the teeth, impacting the measured time series, and about the Sr residence time in the body and the enamel maturation effect of Sr, impacting the predicted time series. To take these uncertainties into account, we used a cross-correlation method to optimize the match between the two times series as a function of the displacement (lag) of measured relative to predicted time series. Resulting lag values were generally small, with a median value of + 41 days (*n* = 11) (SI Appendix, Table S7; Fig. S8). Two larger lags, of + 125 and + 135 days, respectively, were estimated for Kj-369-M3 and Kb-283-M3 (SI Appendix, Table S7), but these lags remained credible considering the potential variation in the DOB and the variation in the development rate of the teeth. In five cases, adding the inferred lag values to the date turned negative correlations between measured and predicted time series into positive ones. In all cases, the correlations were significant (SI Appendix, Table S7; Fig. S8).

**Figure 3:**
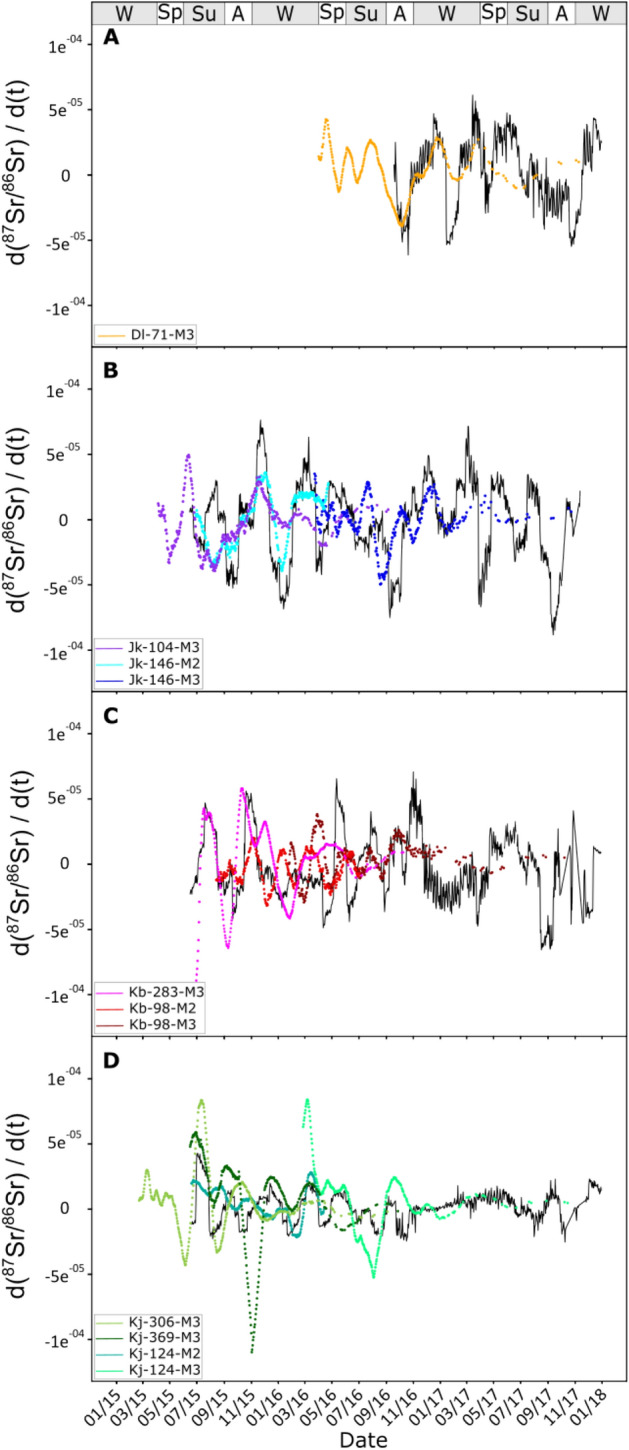
Comparison between the variation of the derivative of the modeling of intra-tooth variations. Variation predicted by the forward model (black line) and the derivative of measured intra-tooth variations of ^87^Sr/^86^Sr running mean (colored full line) for animals belonging to (**A**) Dl, (**B**) Jk, (**C**) Kb, and (**D**) Kj. The time calibration of measured intra-tooth profiles was done using the time lag given by cross-correlation. The date is given as month and year. (W = winter; Sp = spring; Su = summer; A = autumn).

The first derivative of the predicted and measured ^87^Sr/^86^Sr time series showed that most variations are synchronous (Fig. 3). For Dl’s herd, for which there is only one sample, the synchronicity was obvious for the period during which the GPS data were available (Fig. 3A). For Jk’s herd, the synchronicity was also observable for all the samples (Fig. 3B). For other cases, especially for Kb-98, M2 and M3 seem to have recorded identical variations, but they appear to be offset by about 1 month (Fig. 3C). Finally, in Kj’s herd, the synchronicity was good, but variations in the measured time series were larger than those in the predicted ones (Fig. 3D).

## Discussion

The aim of the present work was to evaluate the potential of intra-tooth ^87^Sr/^86^Sr variations to track high-frequency mobility independently assessed using GPS data. Most of the first derivative of the ^87^Sr/^86^Sr variations of the measured and predicted time series showed synchronicity (Fig. 3). The good agreement between the measured and predicted time series suggests that the time averaging model of Passey and Cerling^32^, initially developed for C, is adapted for the reconstruction of ^87^Sr/^86^Sr time series and that ^87^Sr/^86^Sr records are synchronous with those of *δ*^13^C.

It also appears that a better fit would be possible with a better knowledge of the DOB, the tooth formation schedule, and the enamel growth rate. Indeed, in the case of Jk’s herd, some of the variations in the predicted time series seem synchronous with those in the measured time series, but some are likely not (Fig. 3B). This observation holds for the other cases, such as for KB-98, for which the variations recorded by M2 and M3 appear to be offset by about 1 month (Fig. 3C). It is noteworthy that we used a tooth growth model with a constant and a decreasing exponential rate for M2 and M3, respectively. The offset suggests that perhaps this model was an oversimplification.

The existence of synchronicity between the measured and predicted time series allows estimation of the sensibility to detect high-frequency mobility. For instance, during mid-December 2015, Jk’s herd undertook a round trip between two areas (#3 and #4), which differed by about 5000 ppm (0.7137 to 0.7183) in ^87^Sr/^86^Sr (SI Appendix, Table S5; Fig. S6). The duration of their residence in area #4 was approximately 51 days (SI Appendix, Table S5). This movement resulted in a resolvable ^87^Sr/^86^Sr variation of ~ 1000 ppm in the teeth of animals Jk-104 (0.7124 to 0.7135)and Jk-146 (0.7130 to 0.7144) (SI Appendix, Fig. S6). Another example is provided by animal Dl-71 during the winter–spring 2017 nomadization, with a residency lasting 57 and 58 days in each area, respectively (SI Appendix, Table S5). These two examples show that short-term residency of fewer than 2 months can be detected by measuring the ^87^Sr/^86^Sr in tooth enamel. The present results emphasize that considering migrations in terms of distance only is not appropriate, because large Sr isotope differences can be found within a short distance. For example, the same ^87^Sr/^86^Sr variation of 1000 ppm was recorded during winter 2017 in the enamel of two herds (Jk’s and Dl’s) following a nomadization of 14 and 5 km, respectively. It is also noteworthy that all these movements, and those studied in the field of bioarcheology more generally, were accomplished at human walking speed. Forensic science, in contrast, deals with the movements of humans, who (assuming they are not encumbered by a herd), can potentially migrate faster (including by car or by plane). It is therefore even less appropriate to use ^87^Sr/^86^Sr variation as an indication of distance travelled in forensics.

Generally, we found that the measured ^87^Sr/^86^Sr in tooth enamel for a given herd are homogeneous and match the predicted ones. However, two animals displayed unexpectedly low (Kb-98) or high (Kj-369) ratios (SI Appendix, Fig. S6). Their average ^87^Sr/^86^Sr differed by about 2500 ppm from the expected ratios (SI Appendix, Table S6). Moreover, the animals in these two herds (Kb’s and Kj’s) showed significant inter-individual variability. The issue of inter-individual variability has, to our knowledge, been tackled by only two studies^38,39^. In the study of Lewis et al.^39^, the diet was constant and controlled throughout the duration of the experiment. The authors calculated a two standard deviation variability of 100 ppm of the ^87^Sr/^86^Sr in pig tooth enamel for all feeding groups. This number, which represents the minimum variation in the tooth enamel ^87^Sr/^86^Sr that can be expected from individuals raised on identical diets in the same location, is 25 times lower than the difference observed here for Kb-98 and Kj-369. In the study of Anders et al.^38^, the pigs were raised under identical conditions, with the same food, but its composition may have varied over time. For their study, an inter-individual variability of about 2000 ppm in tooth enamel ^87^Sr/^86^Sr can be calculated, which is the order of magnitude found here. It must hence be pointed out that more knowledge is required on the potential—yet poorly constrained—dietary and physiological factors that could contribute to the inter-individual variability in tooth enamel ^87^Sr/^86^Sr of herd animals with similar life histories (e.g., varying contributions of Sr from food *versus* drinking water, differential digestive efficiency or differential bioavailability of potentially heterogeneous Sr-carrying diet components). Ongoing research involving diet-switch experiments in mammal models should help unraveling these factors^40^.

The present study demonstrates that tooth enamel ^87^Sr/^86^Sr time series of caprines can be used to track high-frequency mobility, potentially with a monthly resolution. Despite a precise knowledge of animal mobility and Sr isotope distribution in the landscape, we sometimes found offsets between measured and predicted tooth enamel ^87^Sr/^86^Sr. The role of biological variability also remains to be explained. This could be done by simplified diet shift experiments involving several animals, to take into account potential individual response. For archaeological applications, our results show that the inference of high-frequency mobility using Sr isotope analysis should be treated with an appropriate degree of caution depending on the resolution of the sampling method and the heterogeneity of the bioavailable ^87^Sr/^86^Sr isoscape of the studied area. Diagenesis is an additional caveat for archaeological applications especially when sampling near the enamel-dentine junction. This could be overcome by conducting in situ measurements of trace element concentrations in enamel and co-genetic dentine of the same tooth using LA-ICPMS.

## Materials and methods

### Study area and caprine GPS tracking

The sheep and goats sampled in this study inhabit the district of Nogoonnuur (province of Bayan-Ölgii, Mongolia), on the eastern fringes of the Altai mountain range, at elevations ranging from 1500 m to more than 4000 m (Fig. 1). The climate is strongly continental, with long, cold winters and short, hot summers with extreme temperatures, ranging from + 34 to − 44 °C, and a lower average annual rainfall of 131 mm (meteorological station based at Nogoonnuur village—http://fr.climate.org). Eight caprines (4 sheep and 4 goats) belonging to four different families of Kazakh−Mongolian nomadic pastoralists (shortened here to Dl, Jk, Kb, and Kj), participated in the survey. Caprine mobility was controlled by the herders, and the animals were brought back to the family’s camp every night. Nomadization patterns (seasonal mobility among camps separated by a few km) were monitored precisely using GPS devices fitted on the animals from June 2015 to July 2018 (Globalstar or Iridium collars, Lotek Wireless Inc.—http://www.lotek.com/), programmed to record animal position every 13 and 2 h, respectively (SI Appendix, Table S3). Mobility patterns were described using Lavielle’s segmentation method, which allows determination of changes in animal movement, and discrimination between small-scale movements (within each pasture, in this study) and large-scale movements among pastures, by discriminating series of successive locations (“segments”) during which movements happen at the same spatial scale but between which movements happen at different spatial scales. Due to the nature of the landscape, segmentation was based on altitude, and each segment was considered as a pasture, after visual validation. Only males were sampled, because females may be supplemented with fodder during winter (usually harvested in August and September), potentially overprinting the influence of mobility on enamel ^87^Sr/^86^Sr. Animal slaughter was carried out by the herders themselves for their own consumption. Study animals were slaughtered on two different occasions, during their second or third year: September 2016 (*n* = 4) and November 2017 (*n* = 4), and 11 molars were sampled for analysis (SI Appendix, Table S3). In order to ensure contemporaneity between GPS records and tooth enamel growth, only M3 was sampled on the animals slaughtered in September 2016 and both M2 and M3 were sampled on the animals slaughtered in November 2017.

### Plant sampling to model bioavailable strontium isoscape

In order to build the isoscape of bioavailable Sr, we sampled plants at 156 sites distributed throughout the study area, in September 2016 and November 2017 (Fig. 1 and SI Appendix, Table S1). Sampling localities were selected to cover as much as possible the grazing areas of the animals based on their GPS records, complemented by sites located between grazing areas (SI Appendix, Fig. S1A and B). The distance between neighboring sites was 2.5 km on average, and ranged between 0.2 km and 17.2 km. However, some parts of the study area were difficult to reach due to the presence of an escarpment or the proximity to the Russian border to the north. Each sample represented a mix of plant species present within a circle of ca. 2–3 m diameter. The assemblage was usually dominated by grasses, but also included some forbs and shrubs (SI Appendix, Table S1). In most cases, most collected plant items were plant leaves, but stems, seeds, and fruits were also collected if present. All plant material was air dried and then placed in a loosely sealed paper envelope.

### Solution measurement of the ^87^Sr/^86^Sr in plants

Strontium purification and ^87^Sr/^86^Sr analysis were performed at the Ecole Normale Supérieure in Lyon. Plant samples (*N* = 156) were crushed using a mixer mill and ashed in a muffle furnace at 850 °C for 10 h. For each ashed plant sample, about 5 mg of ashed sample was digested in a mixture of 1 ml of HNO_3_ (15 M) and 1 ml of 30% H_2_O_2_ in a closed Teflon beaker at 120 °C for 8 h. The solution was evaporated and taken up with 500 μL of HNO_3_ (2 M). Strontium was separated from the matrix sample using a column filled with the Sr-spec resin (Eichrom). Following several washes with Milli-Q water and the conditioning of the resin with HNO_3_ (2 M), samples were loaded onto the columns and washed repeatedly with HNO_3_ (2 M), and Sr was eluted with Milli-Q water. The solution was evaporated and taken up with 1 ml of HNO_3_ (0.05 M). Then 100 µl was diluted with 9.9 ml HNO_3_ (0.05 M) marked with 2 ppb of Indium as a calibration and analyzed using a Thermo Scientific iCAP (Thermo Scientific) triple quadrupole inductively coupled plasma mass spectrometer (TQ-ICP-MS) to determine Sr concentrations. Procedural blank levels for plant samples were lower than 0.002 ppb Sr. Finally, samples were diluted with HNO_3_ (0.05 M) to reach 200 ppb Sr concentrations in 2 ml. This final solution was used for isotopic analysis, using a Nu Plasma 1700 (Nu Instruments) multi-collector inductively coupled plasma mass spectrometer (MC-ICP-MS) and, using an invariant normalization ^88^Sr/^86^Sr (8.37521), the exponential fractionation law and a sample-standard bracketing with the NIST SRM-987 reference solution (0.71025 ± 0.00002)^41^. Additional control was carried within the run with standard material NIST SRM-1400 (0.713139 ± 0.000087)^42^ bone ash. The repeated measurements (*N* = 6) of SRM-1400 gave an average ^87^Sr/^86^Sr value of 0.71312 ± 0.000033 (± 2SD). ^88^Sr values (V) obtained for the plant samples were consistent and averaged 0.9642 ± 0.3033 (± 2SD). We tested the potential influence of geographic parameters (i.e. altitude, latitude, and longitude) on plant ^87^Sr/^86^Sr, using linear regression with the function *lm* in the *gstat* package in R (version 3.4.4)^43^*.* Unless noted otherwise, all statistical analyses were conducted using this package, with a threshold of significance at *p-value* < 0.05.

### Spatial modeling of the bioavailable ^87^Sr/^86^Sr isoscape

An ordinary kriging method was used to model the bioavailable ^87^Sr/^86^Sr isoscape. The kriging method provides a spatially explicit interpolation and variance estimate for a given coordinate location, based on a variogram, which is a statistical model of spatial autocorrelation between pairs of sampled points (here, the plant ^87^Sr/^86^Sr). Thus this method allows the spatial behaviour of the data to be taken into account. This variogram model is then used as a basis to interpolate the target variable away from the points. The ^87^Sr/^86^Sr isoscape was performed on a training subsample of 90% (*n* = 140) of the original samples, the remaining 10% (*n* = 16) being saved as a validation subsample, to test model predictions. First, the variogram model was fit with the functions *variogram* (cutoff = 0.1) and *fit.variogram*. The predictive model of bioavailable ^87^Sr/^86^Sr across the study area was then built with the kriging function in ArcGIS 10.0 (Environmental Systems Research Institute), using the parameters of the variogram model (cell size = 1.4 × 10^–3^; SI Appendix, Table S2, Fig. S3A). Moreover, we checked for anisotropy with a directional variogram (Fig. S3B). We tested the reliability of the isoscape by calculating the difference between the field values of the validation subsample and the values predicted in the isoscape for both the training and the validation subsamples (SI Appendix, Fig. S4A and B). We used a normal Q–Q plot to check the distribution of the predicted values (SI Appendix, Fig. S4C). More details are given in the SI Appendix (Supplementary Text).

### Predicted ^87^Sr/^86^Sr intra-tooth variation

The isoscape raster was used to calculate for each herd its predicted daily bioavailable ^87^Sr/^86^Sr, based on the GPS locations of one of its monitored animals (GPS locations of the different animals belonging to the same herd were < 80 m [data not shown], obviating the need to use the location data of the other animals). To this end, for each GPS location covered by the isoscape, we extracted the ^87^Sr/^86^Sr from the raster using the function *extract* (method = “simple”) of the *raster* package in R (version 3.4.4)^44^. For the animals equipped with a Globalstar collar, we kept all the GPS data points (including nighttime data points) to partly make up for the lower recording density. For the other animals, we only kept daylight data points. For each herd, we predicted the daily bioavailable ^87^Sr/^86^Sr by calculating the average of the different bioavailable ^87^Sr/^86^Sr for each day. We then used these daily bioavailable ^87^Sr/^86^Sr to predict a hypothetical intra-tooth ^87^Sr/^86^Sr profile for each of the four herds, using Passey and Cerling’s model^32^. Originally, this model was developed for carbon isotopes in continuously growing teeth, but it has also been used to describe the isotopic time averaging in teeth with definite growth, such as ungulate teeth^11,14,15,37,45^. The main model parameters include the initial mineral content during enamel deposition (*f*_*i*_) and the length of maturation (*l*_*m*_) (corresponding to the length along the tooth over which the maturation stage of the amelogenesis occurs). The model for signal attenuation during enamel formation can be expressed as follows:

1 $$ \delta_{ei} = \left( {f_{i} * \delta_{mi} } \right) + \left( {1 - f_{i} } \right) * \frac{{\sum\nolimits_{n = i + 1}^{{i + 1 + l_{m} }} {\delta_{m} } }}{{l_{m} }} $$

Equation (1) implies that the isotope ratio of fully mineralized enamel at position *i* (*δ*_*ei*_) reflects a contribution from the appositional layer (with *δ*_*mi*_ the initial isotope ratio) and a time-integrated contribution of average isotope ratios of the input signal over the length *l*_*m*_. We set a length of maturation spanning more than 4 months (~ 120 days), as proposed by Zazzo et al.^45^, and a value of 0.25 (*f*_*i*_) for the amount of material incorporated during the secretory stage, based on previous estimates of the mineral content of developing tooth enamel^11,32^. We kept *l*_*m*_ constant for any stage of the crown, hence assuming a constant growth rate of the enamel.

### Laser ablation measurement of the ^87^Sr/^86^Sr in tooth enamel

The teeth were mounted in polyester resin (BROT LAB) and sliced longitudinally with a diamond disc and polished with silicon carbide powder (F500 and F1000; Escil) to expose the entire dental structure. The ^87^Sr/^86^Sr was measured in situ by LA-MC-ICP-MS with a Nu 500 h (Nu Instruments) MC-ICP-MS (multi-collector inductively coupled plasma mass spectrometry), coupled to an Excite (Photon Machines) 193 nm laser ablation system at the Ecole Normale Supérieure in Lyon (SI Appendix, Table S8 and Fig. S9). Sample aerosol was carried to the plasma using a mixture of He and N_2_. Masses 88, 87, 86, 85, 84, and 83 were measured on Faraday cups to resolve the isobaric interference of ^87^Rb on ^87^Sr using ^85^Rb, and of ^84^Kr and ^86^Kr on ^84^Sr and ^86^Sr, respectively, using ^83^Kr. We used a sample-standard bracketing method adapted from the study of Martin et al.^46^, using the sintered "Bone Ash" reference material NIST SRM-1400^47^. The repeated measurements (*N* = 45) of the sintered reference material NIST SRM-1400 gave an average ^87^Sr/^86^Sr value of 0.71384 ± 0.000214 (± 2SD), which is in good agreement with other laser ablation published values^42,46^. The sequence procedure and data processing were adapted from Tacail et al.^48^. Teeth were analyzed in two or three consecutive runs depending on tooth total length. For each sequence, the procedure consisted of the correction of the sample ^87^Sr/^86^Sr with that of the NIST SRM-1400 (0.713139). Finally, a centered moving average on 35 individual measurements (~ 2 mm length of enamel) was used to smooth variability of the final profiles. All data are available in SI Appendix Table S9.

### Statistic comparison among herds and individuals of ^87^Sr/^86^Sr in tooth enamel

In order to compare mean ^87^Sr/^86^Sr between herds, we performed one-way ANOVA tests followed by Tukey’s post-hoc HSD tests. Then, in order to compare inter-individual ^87^Sr/^86^Sr, for each herd, we performed one-way ANOVA tests followed by Tukey’s post-hoc HSD test. We also conducted Bartlett’s test to compare variances between herds and among individuals within a herd.

### Time calibration of measured intra-tooth ^87^Sr/^86^Sr profiles

To compare predicted ^87^Sr/^86^Sr time series with measured intra-tooth ^87^Sr/^86^Sr profiles, it was necessary to associate the position in the tooth with the approximate date in the calendar when this part of the tooth was formed. To do this, it is necessary to know the DOB of the animal, as well as the dental development rate and the growth rate of its molars. However, none of these parameters are known with certainty. Interviews with herders indicated that the season of birth is very restricted, extending from February to April, with slight variations between years and herders, which is in accordance with Fijn^49^. In the absence of individual information, we chose an arbitrary DOB of March 31 for all the animals. This clearly introduces an uncertainty of ± 1 month in the predicted tooth enamel ^87^Sr/^86^Sr time series. We calculated the onset of mineralization of M2 and M3 to be, on average, at 1.3 months and 10.3 months after birth, respectively, based on literature values for caprines (SI Appendix, Table S10). We used these average dental development rates (Table S10) to calculate the duration of formation for each M2 and M3, depending on the state of crown formation (SI Appendix, Table S11).

Finally, we calculated individual tooth growth rates specifically for M2 and M3. For M2, we calculated a constant growth rate (τ) by dividing tooth length (L_max_) by the duration of tooth formation (SI Appendix, Table S10). To convert measured distances along the tooth into time (*t*_*d*_) for any point at a distance *d* from the enamel–root junction in M2, we used the following equation:

2 $$ t_{d} = t_{0} + \left( {\frac{{L_{\max } - d}}{\tau }} \right) $$

For M3, tooth growth rate may decrease exponentially^37^. Therefore, to convert measured distances along the tooth into time (*t*_*d*_) for any point at a distance *d* from the enamel–root junction in M3, we used the following equation:

3 $$ t_{d} = t_{0} + 1/\lambda * \ln \left( {\frac{{L_{\max } }}{d}} \right) $$

where λ is the rate constant of the growth (λ = ln(2)/t_1/2_), t_1/2_ is the time taken for the tooth to grow to half of its maximum length (*L*_*max*_), and t_0_ is the time at which the tooth begins to grow. Thus, t_0_ depends on the DOB and the onset of formation of M3. We set t_1/2_ to 141 days^37^. Given the logarithmic characteristics of the model, the last 2–3 mm gave dates tending well beyond the date of the individual’s death, as such these were omitted.

### Cross-correlation of time series

Because of the unavoidable imprecision associated with the time calibration of intra-tooth ^87^Sr/^86^Sr profiles, there may be a temporal shift between the measured and predicted time series. To detect this shift and readjust the two time series, we used the function *ccf* (cross-correlation function)^50^. This function calculates the correlation between two time series for various time lags between them, allowing inferring of the existence and amount of temporal shift between time series. Then, we calculated the Pearson’s correlation coefficient and the linear regression between the ^87^Sr/^86^Sr of the measured and predicted times series with the selected lag value. Finally, we also visually evaluated the amount of synchronicity of the readjusted ^87^Sr/^86^Sr of the measured and predicted time series, by calculating the first derivatives of the ^87^Sr/^86^Sr (*d*(^87^Sr/^86^Sr)/*dt*), smoothed on 30 days, for each time series, to estimate the slope of the variation.

## Supplementary Information

Supplementary Information 1.

Supplementary Table S1.

Supplementary Table S9.

## Data Availability

All data needed to evaluate the conclusions in the paper are present in the paper and/or the Supplementary Materials. Additional data related to this paper may be requested from the authors.
