# Supplementary material for: Monthly mobility inferred from isoscapes and laser ablation strontium isotope ratios in caprine tooth enamel
Source: Sci Rep. 2021 Jan 26;11:2277. doi: 10.1038/s41598-021-81923-z (PMC7838167; doi:10.1038/s41598-021-81923-z)
Supplement: Supplementary file 1 — Supplementary Information 1. [file 41598_2021_81923_MOESM1_ESM.docx]

Supplementary Information for

**Monthly mobility inferred from isoscapes and laser ablation strontium isotope ratios in tooth enamel**

N. Lazzerini, V. Balter, A. Coulon, T. Tacail, C. Marchina, M. Lemoine, N. Bayarkhuu, Ts. Turbat, S. Lepetz, A. Zazzo*

* Corresponding author Email: antoine.zazzo@mnhn.fr

**This PDF file includes:**

Supplementary text

Figures S1 to S9

Tables S1 to S11

Supplementary Information Text

**Results of the spatial modelling of the bioavailable ^87^Sr/^86^Sr isoscape by ordinary kriging method**

A kriging method was used to create the strontium isoscape. Kriging is a geostatistical interpolation method based on a variogram, a statistical model of spatial autocorrelation between pairs of sampled points (here, the 156 plant ^87^Sr/^86^Sr). The variogram model was used to interpolate (predict) the value of the modelled variable (^87^Sr/^86^Sr) in between sample sites. We used ordinary kriging to estimate patterns of ^87^Sr/^86^Sr variation.

The semivariogram (Fig. S3A) showed that the plant ^87^Sr/^86^Sr values were isotropic, following an exponential model (Table S2). There was a small mean error (−2.5E^-05^) between predicted and measured ^87^Sr/^86^Sr values of training subsamples, with a good correlation between measured and predicted values (Fig. S4A), indicating unbiased prediction errors, with a weak overprediction of small values and a weak underprediction of larger values. The root mean-square error was also low (0.0005) and not different from the standard error (SE) of the estimate (0.0005), indicating a robust assessment of variability in the model predictions.

The corresponding Q–Q plot was used to compare the shape of the distribution of predicted values and measured values from the isoscape (Fig. S4B). The straight line represents the hypothetical case where the two distributions are the same. The linearity of the points suggests that the predicted data are normally distributed.

The robust assessment by the model predictions was confirmed by the small absolute difference between the ^87^Sr/^86^Sr values of the validation subset of plant samples (N = 16) removed from the original dataset to build the isoscape and model predictions at their locations (mean = 0.00022, range = −0.0022 to 0.0031). The validation subsample showed a good correlation between predicted and measured values but with larger overprediction of small values and larger underprediction of larger values than for the training subsample (Fig. S4C).

Supplementary figures


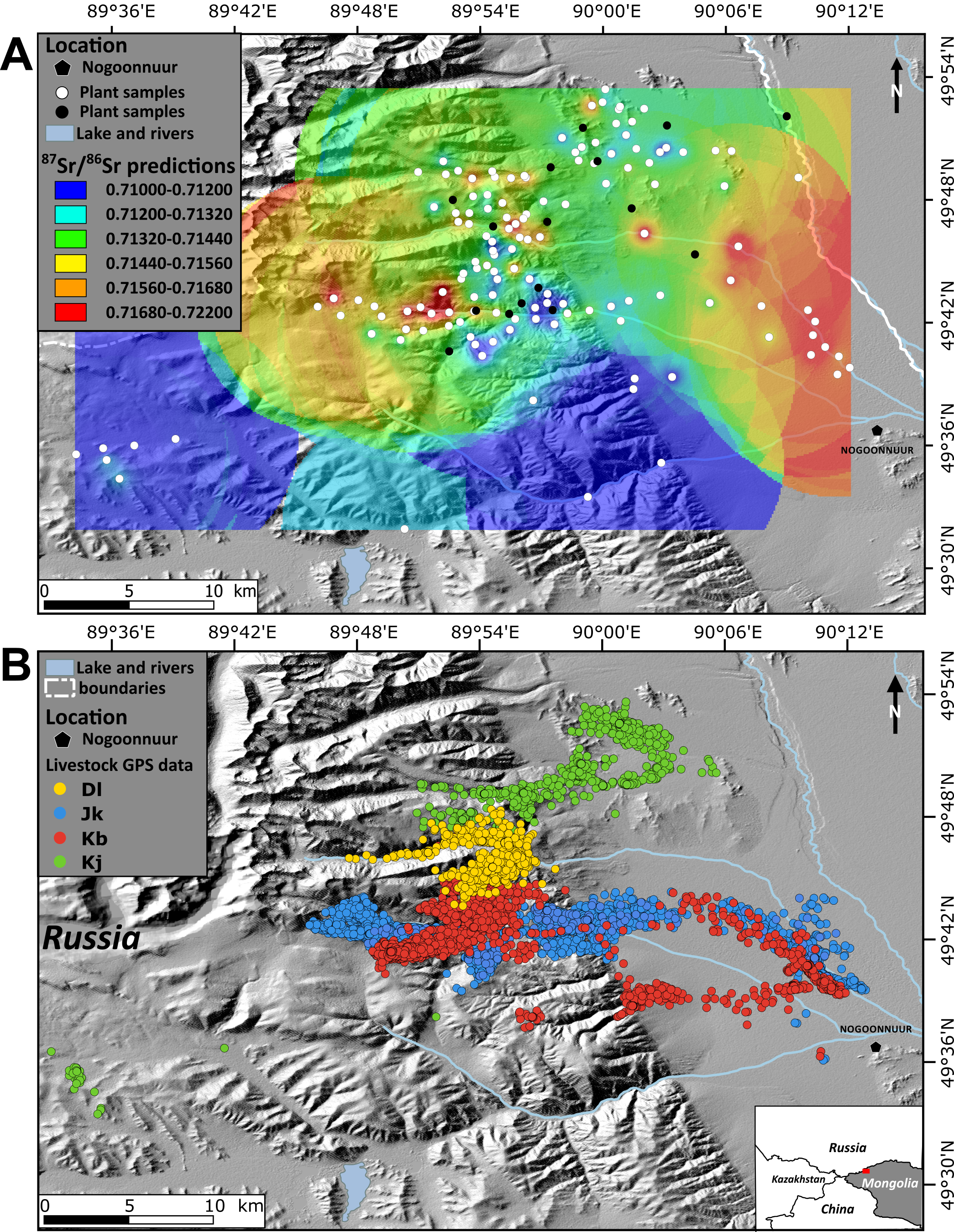


Fig. S1. Plant sampling and GPS locations. Plant sampling (A) and GPS-monitored livestock locations (B). In panel (B), each color corresponds to a different herder (Dl, Jk, Kb, or Kj).


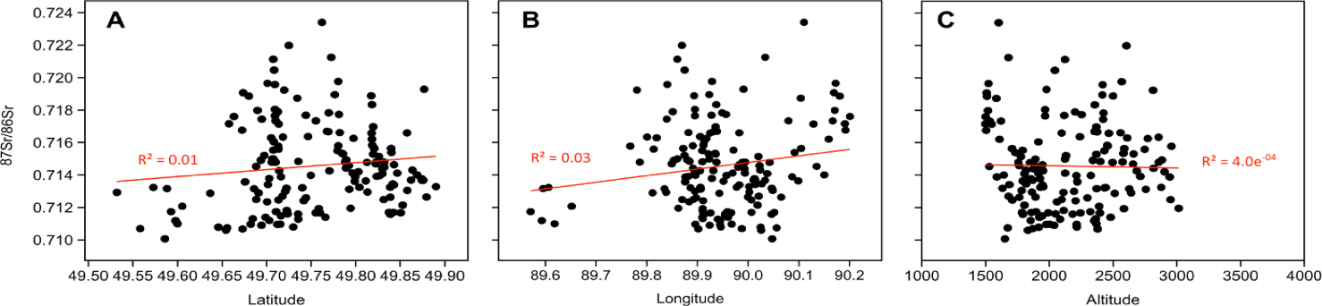


Fig. S2. Bioavailable ^87^Sr/^86^Sr values from the sampling localities. Plotted relative to (A) latitude, (B) longitude, and (C) altitude.


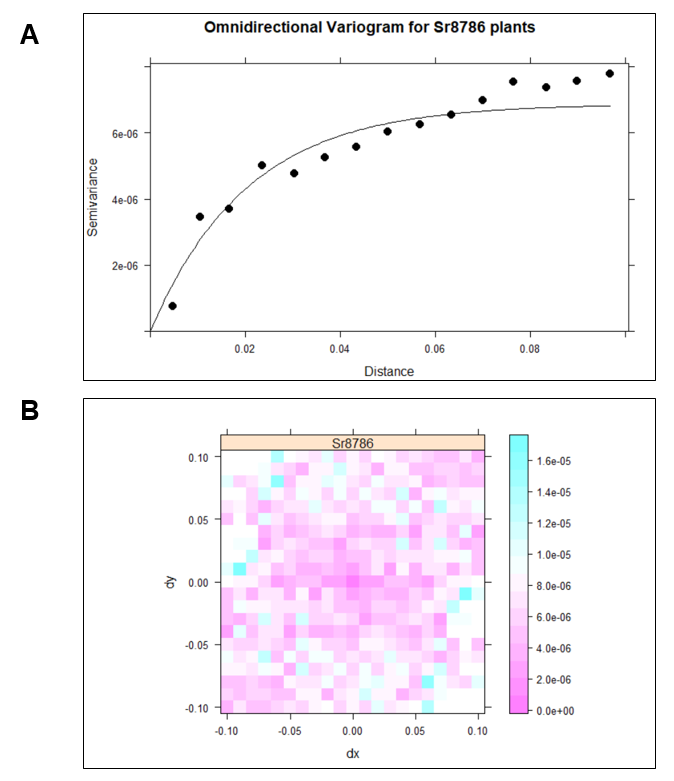


Fig. S3. Spatial variation ^87^Sr/^86^Sr values of sampled plants. (A) Semivariogram of ^87^Sr/^86^Sr values of plants. An exponential model on short distances was the best fit for the data. (B) The directional “map,” computed with the Gstat package in R, showing the spatial variation of ^87^Sr/^86^Sr (color scale) in x and y (km) from a point of origin (0;0).


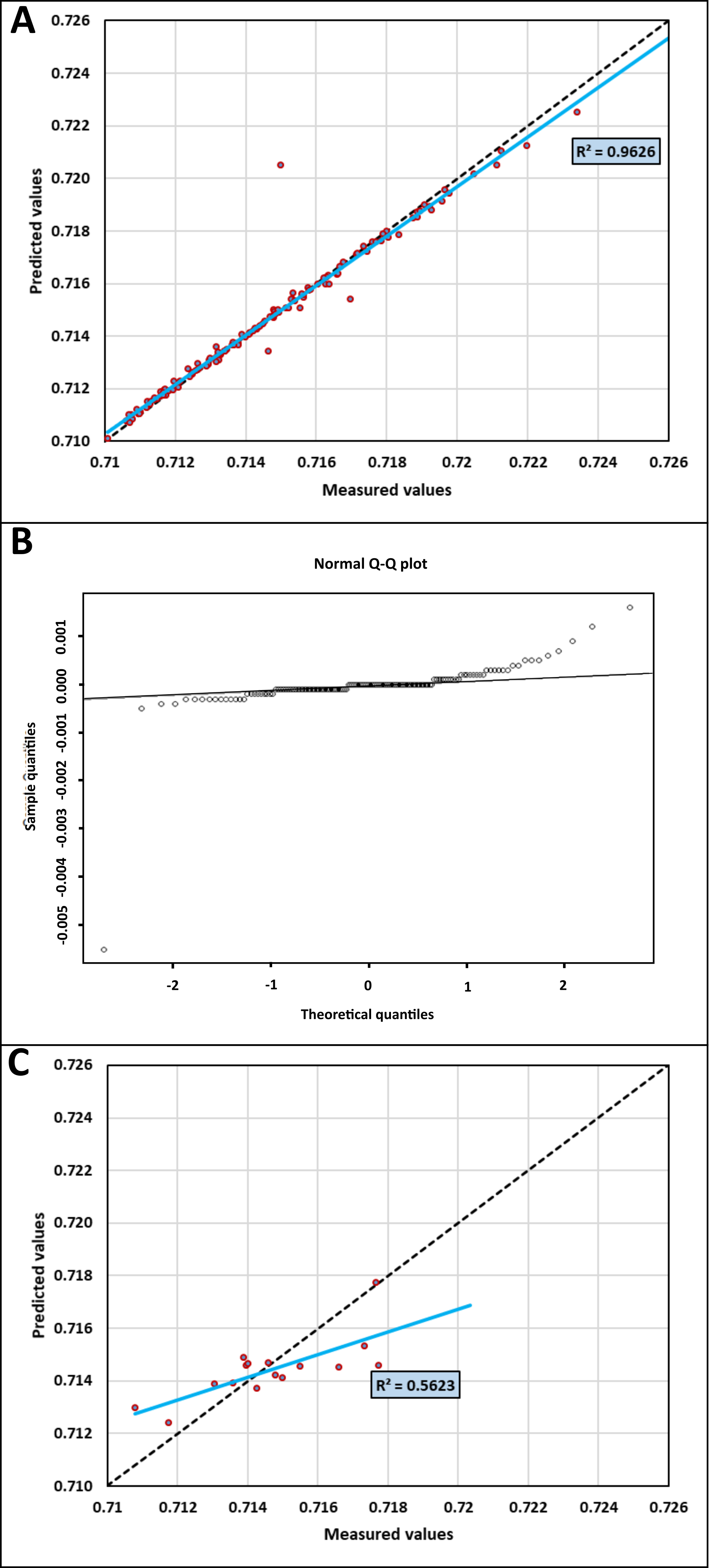


Fig. S4. Predicted versus measured ^87^Sr/^86^Sr values of plants. (A) Scatter plot showing predicted values (Y-axis) from the isoscape versus measured values (X-axis) of bioavailable ^87^Sr/^86^Sr from the 140 training sampling sites. The black line is the 1:1 regression line, whereas the blue line is the fitted regression line (B) Q–Q plot for the training subsample, showing the standardized errors on the Y-axis (quantiles of the difference between the predicted and measured data values) and normal values on the X-axis, subdivided by their corresponding quantiles. The gray line represents a normal distribution. (C) Scatter plot showing predicted values (Y-axis) from the isoscape versus measured values (X-axis) of bioavailable ^87^Sr/^86^Sr from the 16 validation subsample sites. The black line is the 1:1 regression line, whereas the blue line is the fitted regression line, showing overprediction of small values and underprediction of larger values.


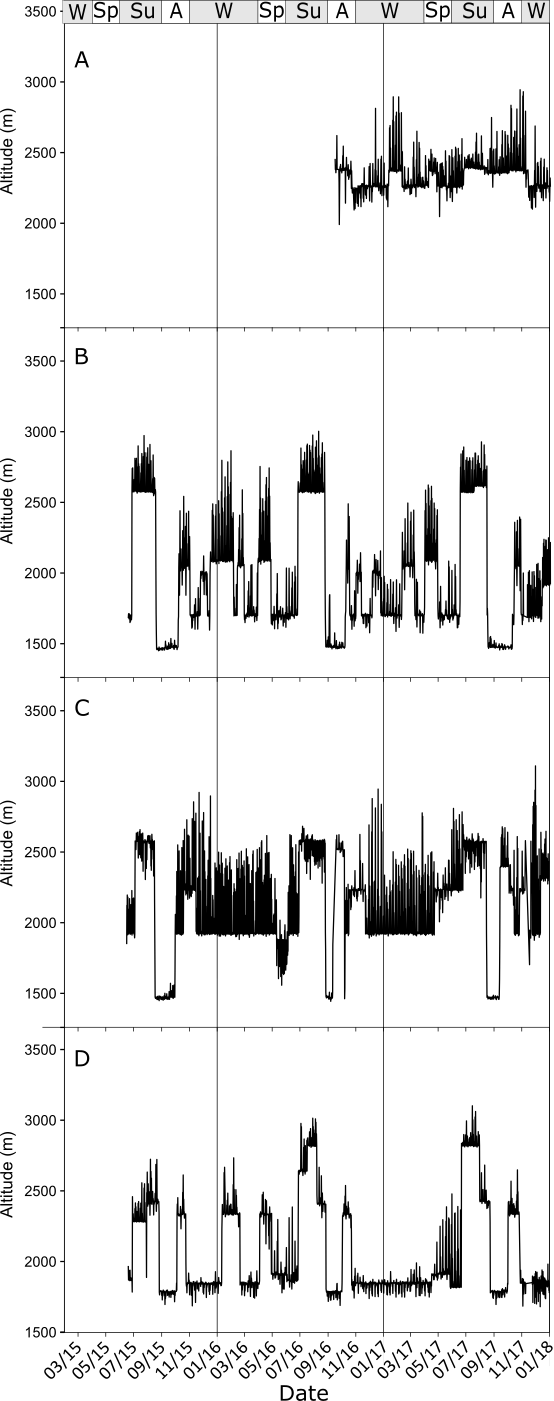


Fig. S5. Temporal altitudinal variation of GPS-monitored caprines. Animals belonging to the herd of (A) Dl, (B) Jk, (C) Kb, and (D) Kj. The animals herded by Dl show a lower variability of altitudinal mobility than those of the other herders. Vertical lines represent the change of year (2015/2016/2017). (W = winter; Sp = spring; Su = summer; A = autumn.)

**
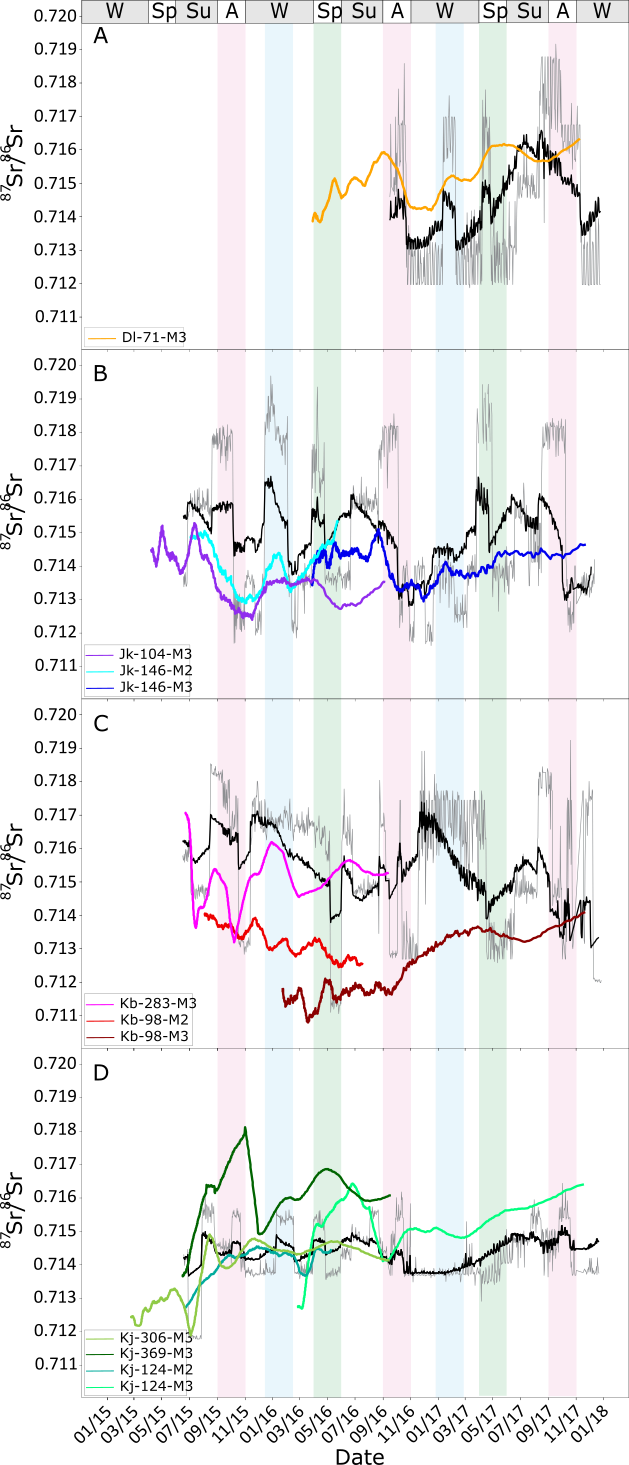
**

Fig. S6. Temporal variation in published, predicted, and measured ^87^Sr/^86^Sr values. Temporal variation in bioavailable ^87^Sr/^86^Sr values of pastures grazed by cattle (gray line); intra-tooth variation predicted by Passey and Cerling’s model (*1*) (thick black line); and measured intra-tooth variation in ^87^Sr/^86^Sr running mean (colored full line) for animals belonging to (A) Dl, (B) Jk, (C) Kb, and (D) Kj. The time calibration of measured intra-tooth profiles was done using the time lag given by cross-correlation (Table S7). (W = winter; Sp = spring; Su = summer; A = autumn.)


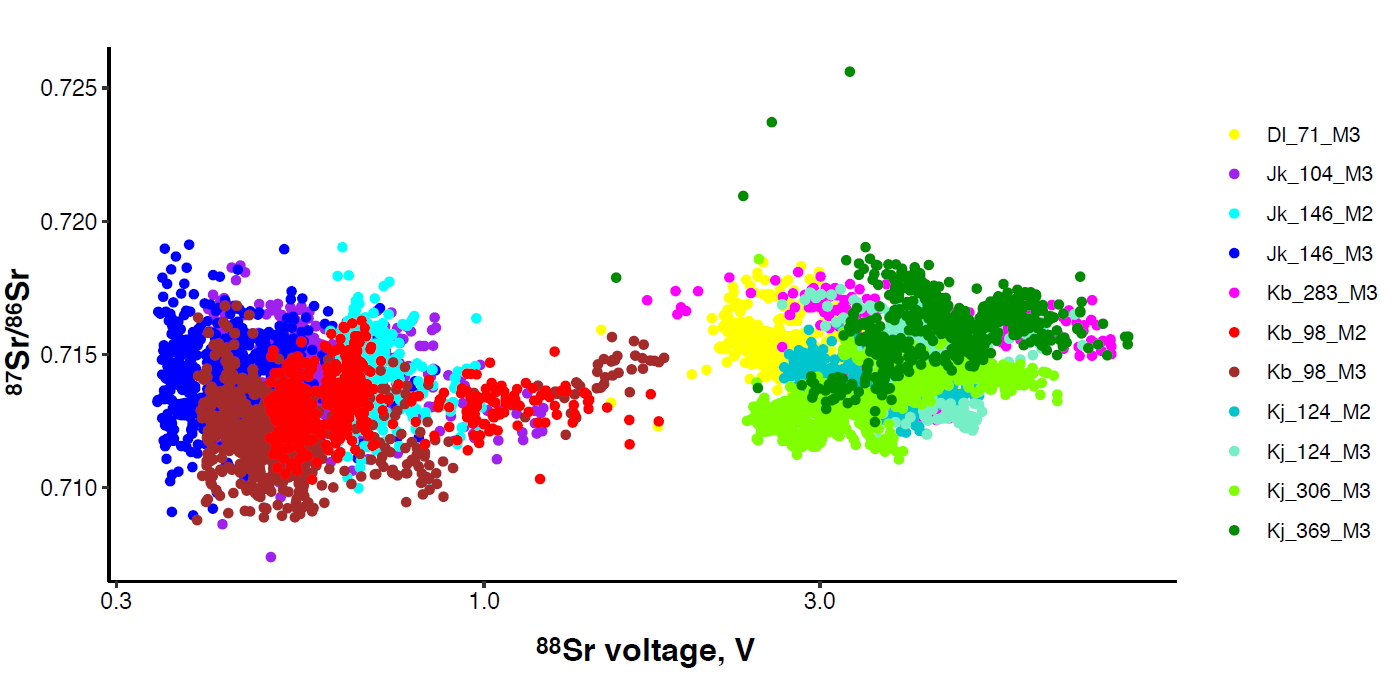


**Fig. S7**. Measured ^87^Sr/^86^Sr values plotted as a function of ^88^Sr voltage for each tooth. Data available in **Table S9**)


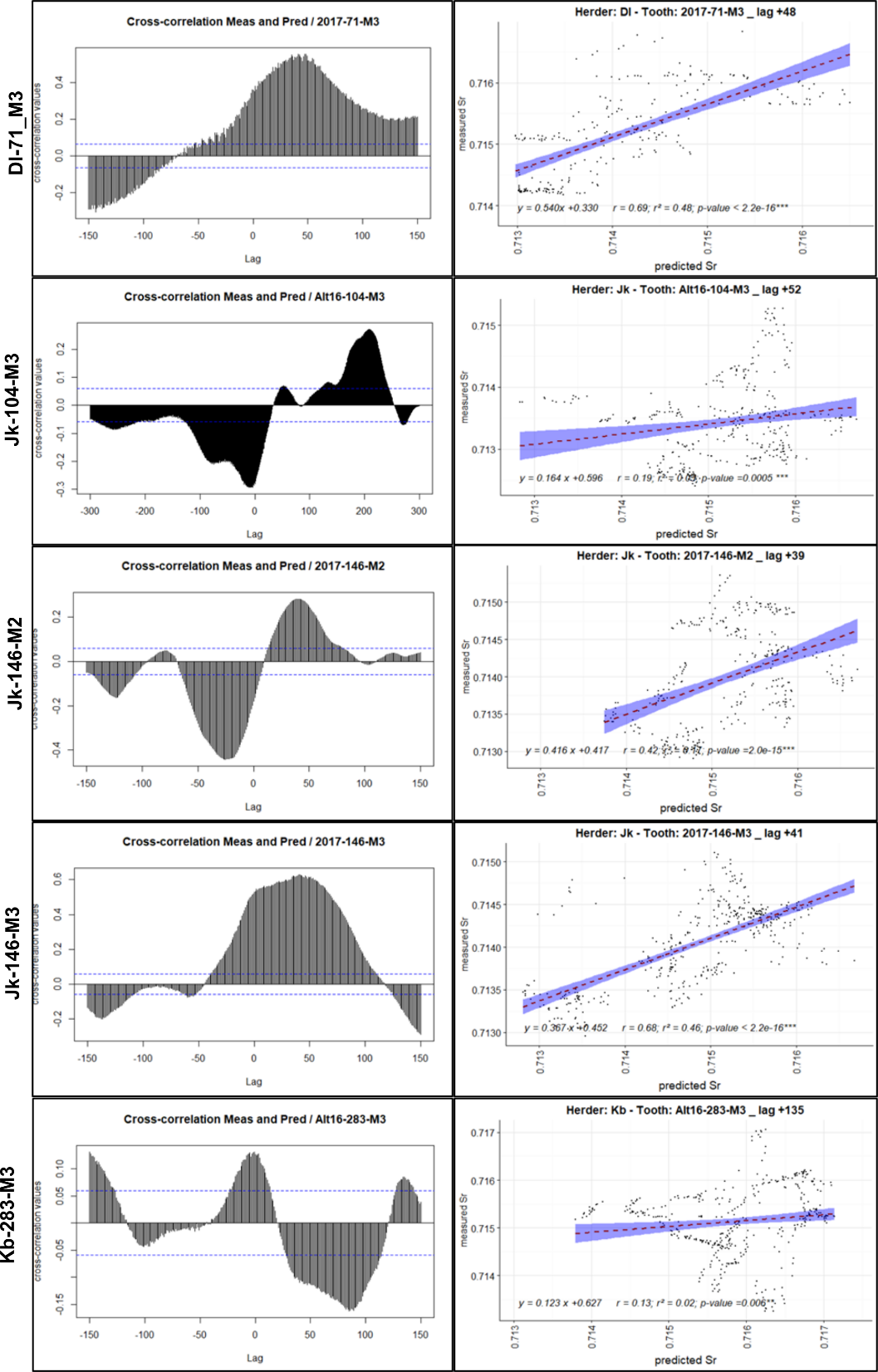

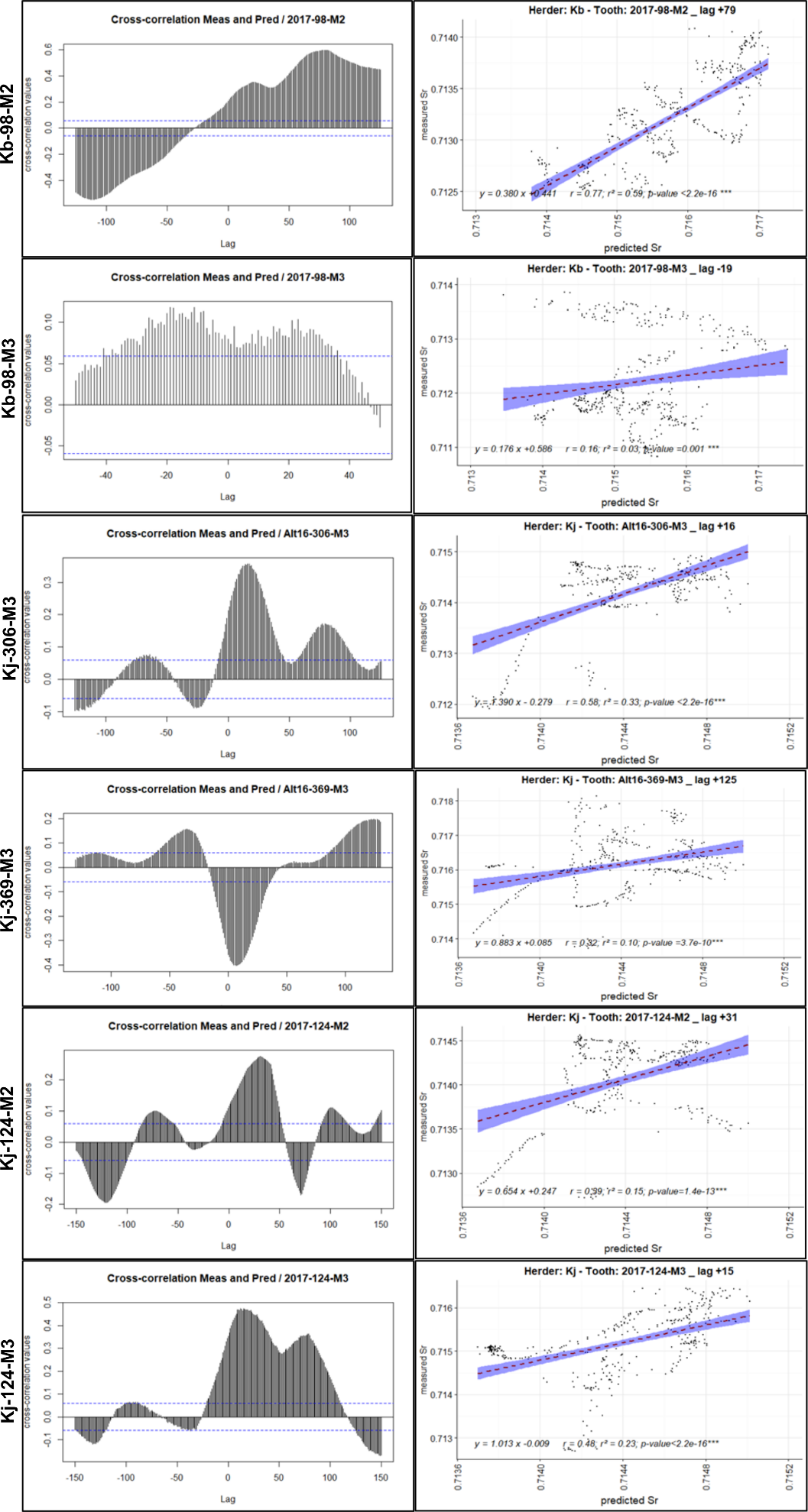


Fig. S8. Predicted intra-tooth ^87^Sr/^86^Sr values as a function of the lag and correlation with measured values. For each tooth, the graph on the left shows ccf (cross-correlation function) values for predicted intra-tooth ^87^Sr/^86^Sr values in function of the lag of measured intra-tooth ^87^Sr/^86^Sr values. The chosen lag values correspond to the maximum cross-correlation function (*ccf*) value. For each tooth, the graph on the right shows correlation between measured and predicted intra-tooth ^87^Sr/^86^Sr profiles for each tooth when the inferred time lag (Table S7) is applied to time calibration of measured intra-tooth ^87^Sr/^86^Sr profiles.


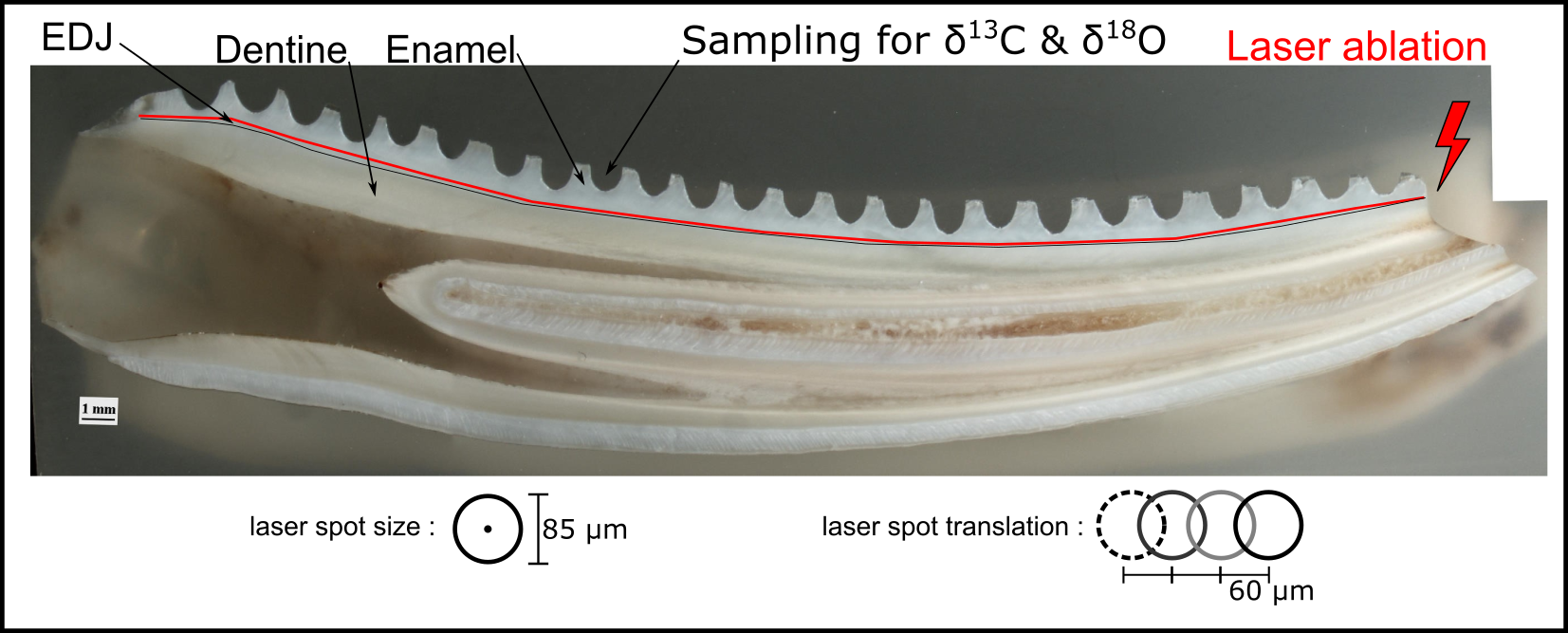


Fig. S9. Photo of tooth crown included in polyester resin (2017-146-M3) depicting the laser ablation track and features of the spot size.

Supplementary Tables

Table S1. Taxonomic composition, location, and biologically available ^87^Sr/^86^Sr of the 156 plant samples, with associated standard error (SE). (See Excel file provided in supplementary material).

Table S2. Parameters of the geospatial modelling, characteristics, and accuracy of the kriging model.

| **Model parameters** |  |
| --- | --- |
| Kriging type | Ordinary |
| Semivariogram | Exponential |
| Major range | 0.019338 |
| Partial sill | 0.000007219 |
| Nugget | 0 |
| Lag size | 0.01 |
| Output cell size | 1.43235.10^-3^ |
| Search radius | Fixed |
| Distance | 0.1 |
| **Kriging results** |  |
| Number of samples | 140 |
| Mean error | -2.51e^-05^ |
| Root mean square error (RMSE) | 0.0005 |
| Mean absolute percentage error (MAPE) | 0.387251 |
| Residual standard error | 0.0005 |
| Standard deviation of predicted values | 0.0026 |
| Standard deviation of error | 0.0005 |
| **Results of the reliability assessment of the kriging model** |  |
| Number of samples | 16 |
| Mean error | 0.00022 |
| Residual standard error | 0.0008 |
| Standard deviation of error | 0.0016 |
| Root mean square error (RMSE) | 0.0149 |
| Maximum error | 0.0031 |
| Minimum error | -0.0022 |

Table S3. Information about animals fitted with GPS collars and teeth sampled for ^87^Sr/^86^Sr analysis. DOD = date of death. GPS type is either Globalstar© (Gb – recording every 13 hours) or Iridium© (Ir – recording every 2 hours). ^*^Age estimated after Payne (*2*) and Jones (*3*). † GPS out of service before the death of the animal; we used GPS data from another collar in the same herd to complete the location data.

| **Sample** | **DOD** | **Year of birth** | **Approximate age**  **at death (months)*** | **Species** | **Sex** | **Tooth**  **sampled** | **GPS type** | **Start GPS** | **End GPS** | **Herder** |
| --- | --- | --- | --- | --- | --- | --- | --- | --- | --- | --- |
| Alt16-104 | 05/09/2016 | 2014 | 30 | *Ovis aries* | M | M_3_ left | Gb | 19/06/2015 | 07/09/2016 | Jk |
| Alt16-283 | 13/09/2016 | 2014 | 30 | *Ovis aries* | M | M_3_ left | Gb | 16/06/2015 | 12/09/2016 | Kb |
| Alt16-306 | 14/09/2016 | 2014 | 30 | *Ovis aries* | M | M_3_ left | Ir | 19/06/2015 | 14/09/2016 | Kj |
| Alt16-369 | 21/09/2016 | 2014 | 30 | *Capra hircus* | M | M_3_ left | Gb | 19/06/2015 | 20/09/2016 | Kj |
| 2017-71 | 17/11/2017 | 2015 | 32 | *Capra hircus* | M | M_2_ & M_3_ left | Gb | 16/09/2016 | 17/11/2017 | Dl |
| 2017-98 | 20/11/2017 | 2015 | 32 | *Capra hircus* | M | M_2_ & M_3_ left | Gb | 18/09/2016 | 09/11/2017 | Kb |
| 2017-124 | 24/11/2017 | 2015 | 32 | *Ovis aries* | M | M_2_ & M_3_ left | Gb | 21/09/2016 | 09/11/2017 | Kj |
| 2017-146 | 23/11/2017 | 2015 | 32 | *Capra hircus* | M | M_2_ & M_3_ left | Gb | 05/09/2016 | 03/05/2017 † | Jk |

Table S4. Mobility patterns of four herders as inferred from domestic caprine monitoring, and characteristics of the GPS monitoring. For each herder, the mobility patterns were estimated based on the data from 1 to 3 animals (see Table S3). N = number. SD = standard deviation. A one-way ANOVA with Tukey’s post-hoc HSD was used to compare the mean altitude between herds. *** = the average altitude of the herds is significantly different from that of all other herds. Detailed mobility patterns with camps are provided in Table S5.

| **Herder identifier** | | **Dl** | **Jk** | **Kb** | **Kj** |
| --- | --- | --- | --- | --- | --- |
| **Start GPS** | | 16/09/2016 | 19/06/2015 | 16/06/2015 | 19/06/2015 |
| **End GPS** | | 17/11/2017 | 04/11/2017 | 09/11/2017 | 09/11/2017 |
| **N days** | | 427 | 869 | 877 | 874 |
| **N GPS data** | | 1239 | 1599 | 1608 | 1606 |
| **Altitude (m.asl)** | mean ± SD | 2364 ± 130*** | 1980 ± 420******* | 2127 ± 339******* | 2076 ± 338******* |
|  | max | 3002 | 3005 | 2946 | 3102 |
|  | min | 1990 | 1451 | 1453 | 1684 |
| **N camps** | | 5 | 8 | 10 | 11 |
| **N nomadisation** | | 9 | 31 | 21 | 23 |
| **N nomadisation/year** | | 7.7 | 13.0 | 8.7 | 9.6 |
| **Distance travelled (km)** | | 35 | 513 | 327 | 318 |
| **Distance traveled/year (km)** | | 30 | 216 | 136 | 133 |
| **Average distance travelled for each nomadization (km)** | | 3.9 ± 1.7 | 16.5 ± 12.8 | 15.6 ± 14.0 | 14.1 ± 15.9 |
| **Average grazing area by pasture (km²)** | | 12.5 | 22.3 | 18.9 | 19.6 |
| **N days on pasture** | mean ± SD | 50 ± 27 | 28 ± 17 | 40 ± 44 | 39 ± 33 |
|  | max | 90 | 59 | 178 | 175 |
|  | min | 18 | 4 | 9 | 20 |
| P < 0.1 = . | |  |  |  |  |
| P < 0.05 = * | |  |  |  |  |
| P < 0.01 = ** | |  |  |  |  |
| P < 0.001 = *** | |  |  |  |  |

Table S5. Calendar mobility of each herder.

Table S6. Summary results of measured intra-tooth ^87^Sr/^86^Sr values. Moving average on 35 individual measurements (top panel) and summary results of bioavailable (middle panel) and predicted (bottom panel) ^87^Sr/^86^Sr time series.

| **Tooth** | **Herder** | **^87^Sr/^86^Sr ratio** | | | | | |
| --- | --- | --- | --- | --- | --- | --- | --- |
|  |  | **Mean** | **2SD** | **Max** | **Min** | **Amplitude** | **N** |
| Alt16-104-M3 | Jk | 0.71381 | 0.00145 | 0.71530 | 0.71240 | 0.00289 | 664 |
| Alt16-283-M3 | Kb | 0.71509 | 0.00164 | 0.71708 | 0.71321 | 0.00388 | 670 |
| Alt16-306-M3 | Kj | 0.71346 | 0.00183 | 0.71492 | 0.71189 | 0.00303 | 686 |
| Alt16-369-M3 | KJ | 0.71602 | 0.00198 | 0.71814 | 0.71366 | 0.00447 | 515 |
| 2017-71-M3 | Dl | 0.71499 | 0.00139 | 0.71716 | 0.71382 | 0.00334 | 644 |
| 2017-98-M2 | Kb | 0.71322 | 0.00087 | 0.71409 | 0.71246 | 0.00162 | 429 |
| 2017-98-M3 | Kb | 0.71200 | 0.00158 | 0.71422 | 0.71081 | 0.00340 | 630 |
| 2017-124-M2 | Kj | 0.71406 | 0.00094 | 0.71456 | 0.71271 | 0.00185 | 365 |
| 2017-124-M3 | Kj | 0.71507 | 0.00185 | 0.71656 | 0.71269 | 0.00375 | 547 |
| 2017-146-M2 | Jk | 0.71400 | 0.00132 | 0.71536 | 0.71290 | 0.00246 | 620 |
| 2017-146-M3 | Jk | 0.71414 | 0.00093 | 0.71511 | 0.71296 | 0.00216 | 494 |
| **Bioavailable** | **Herd** |  |  |  |  |  |  |
|  | Dl | 0.71452 | 0.00393 | 0.71916 | 0.71179 | 0.00737 | 672 |
|  | Jk | 0.71507 | 0.00421 | 0.72062 | 0.71162 | 0.00900 | 902 |
|  | Kb | 0.71529 | 0.00386 | 0.71926 | 0.71106 | 0.00820 | 1104 |
|  | Kj | 0.71447 | 0.00169 | 0.71645 | 0.71178 | 0.00467 | 1116 |
| **Predicted** | **Herd** |  |  |  |  |  |  |
|  | Dl | 0.71450 | 0.00185 | 0.71657 | 0.71298 | 0.00359 | 562 |
|  | Jk | 0.71496 | 0.00176 | 0.71670 | 0.71281 | 0.00389 | 902 |
|  | Kb | 0.71531 | 0.00198 | 0.71740 | 0.71304 | 0.00437 | 984 |
|  | Kj | 0.71446 | 0.00079 | 0.71521 | 0.71367 | 0.00153 | 995 |

Table S7. Results from cross-correlation for each tooth with maximum cross-correlation function (*ccf*) values, and corresponding time lag (in days) that was applied to time-calibrate each tooth.

| **Herder** | **Tooth** | **Maximum *ccf* value** | **lag value** | **Pearson’s correlation coefficient between measured and predicted intra-tooth ^87^Sr/^86^Sr values** |
| --- | --- | --- | --- | --- |
| Dl | 2017-71-M3 | *ccf* = 0.554 | lag = +48 | R = 0.69; p-val < 2.2^e^-16 |
| Kb | Alt16-283-M3 | *ccf* = 0.085 | lag = +135 | R = 0.13; p-val =0.006 |
| Kb | 2017-98-M2 | *ccf* = 0.321 | lag = +79 | R = 0.77; p-val < 2.2^e^-16 |
| Kb | 2017-98-M3 | *ccf* = 0.118 | lag = −19 | R = 0.16; p-val =0.001 |
| Jk | Alt16-104-M3 | *ccf* = 0.071 | lag = +52 | R = 0.19; p-val =0.0005 |
| Jk | 2017-146-M2 | *ccf* = 0.283 | lag = +39 | R = 0.42; p-val = 2.0^e^-15 |
| Jk | 2017-146-M3 | *ccf* = 0.630 | lag = +41 | R = 0.68; p-val < 2.2^e^-16 |
| Kj | Alt16-306-M3 | *ccf* = 0.358 | lag = +16 | R = 0.58; p-val < 2.2^e^-16 |
| Kj | Alt16-369-M3 | *ccf* = 0.197 | lag = +125 | R = 0.32; p-val = 4^e^-10 |
| Kj | 2017-124-M2 | *ccf* = 0.276 | lag = +31 | R = 0.39; p-val =1.4^e^-139 |
| Kj | 2017-124-M3 | *ccf* = 0.475 | lag = +15 | R = 0.48; p-val < 2.2^e^-16 |

Table S8. Parameters and operating conditions for Sr isotope determination in tooth samples by laser ablation MC-ICPMS.

| **Parameters** | **Values** |
| --- | --- |
| ***Laser ablation 193 nm Excite*** |  |
| Wavelength | 193 nm |
| He flow rate | 0.7 L/min |
| ***Ablation*** |  |
| Spot size | 85 μm |
| Frequency | 10 Hz |
| Fluence | 15.18 J/cm² |
| Sampling scheme | Line (dynamic) |
| Line translation rate | 60 μm/s |
| Sample line length | ~ 18 mm |
| Standard line length | 3 mm |
| ***MC-ICPMS Nu 500 HR*** |  |
| *Argon cool gas flow rate* | 14 L/min |
| Auxillary gas glow | 1.4 L/min |
| Sample gas flow | 0.7 L/min |
| Plasma power | 1350 |
| Resolution |  |
| ***Data collection*** |  |
| Gas background | 30 s |
| Sample | ~ 300 s |
| Integration | 2 s |

Table S9. ^87^Sr/^86^Sr rom laser ablation along each tooth crown of the study, with associated moving average and standard deviation (SD) of the moving average. (See Excel file provided in supplementary material).

Table S10. Caprine dental development rates (in months after birth) from four published datasets.

| **Reference** | **Start M2** | **End M2** | **Start M3** | **End M3** |
| --- | --- | --- | --- | --- |
| Milhaud & Nézit 1991 (*4*) | 0 | 12 | 8 | 22 |
| Upex et al. 2012 (*5*) | 2 | 16 | 14 | 33 |
| Weinreb & Sharav 1964 (*6*) | 2 | 12 | 11 | 25 |
| Zazzo et al. 2010 (*7*) | 1 | 12 | 8 | 24 |
| **Average** | **1.3** | **13.0** | **10.3** | **26.0** |

Table S11. Dental development of the caprines in this study. Teeth are classified as “complete” when root development has begun on the posterior buccal cusp; as “almost complete” when root development has begun on the other cusps but not yet on the posterior buccal cusp; and as “incomplete” when root development has not begun on the cusps.

| **Individual** | **Known age at death (years)** | **Second molar** | **Third molar** |
| --- | --- | --- | --- |
| 2017-71 | 2.7 | Complete | Almost complete |
| 2017-98 | 2.7 | Complete | Complete |
| 2017-124 | 2.7 | Complete | Complete |
| 2017-130 | 2.7 | Complete | Almost complete |
| 2017-146 | 2.7 | Complete | Complete |
| 2016-104 | 2.5 | Complete | Incomplete |
| 2016-283 | 2.5 | Complete | Incomplete |
| 2016-306 | 2.5 | Complete | Almost complete |
| 2016-369 | 2.5 | Complete | Incomplete |

SI References

1. B. H. Passey, T. E. Cerling, Tooth enamel mineralization in ungulates: implications for recovering a primary isotopic time-series. *Geochim. Cosmochim. Acta*. **66**, 3225–3234 (2002).

2. S. Payne, Kill-off Patterns in Sheep and Goats: the Mandibles from Aşvan Kale. *Anatol. Stud.* **23**, 281–303 (1973).

3. G. G. Jones, in *Recent advances in ageing and sexing animal bones* (Oxbow Books, 2006), pp. 155–178.

4. G. Milhaud, J. Nezit, Développement des molaires chez le mouton. Etude morphologique, radiographique et microdurométrique. *Recl. Médecine Vét.* **167**, 121–127 (1991).

5. B. Upex, K. Dobney, Dental enamel hypoplasia as indicators of seasonal environmental and physiological impacts in modern sheep populations: a model for interpreting the zooarchaeological record. *J. Zool.* **287**, 259–268 (2012).

6. M. M. Weinreb, Y. Sharav, Tooth development in sheep. *Am. J. Vet. Res.* **25**, 891–908 (1964).

7. A. Zazzo, M. Balasse, B. H. Passey, A. P. Moloney, F. J. Monahan, O. Schmidt, The isotope record of short- and long-term dietary changes in sheep tooth enamel: Implications for quantitative reconstruction of paleodiets. *Geochim. Cosmochim. Acta*. **74**, 3571–3586 (2010).
